# 70. Impact of the Accelerate Pheno™ System on Clinical and Antimicrobial Outcomes among Inpatients with Gram-Negative Bacteremia at a 528-bed Community Teaching Hospital

**DOI:** 10.1093/ofid/ofab466.272

**Published:** 2021-12-04

**Authors:** William P DePasquale, Mary L Staicu, Sean Stainton, Maryrose R Laguio-Vila, Mindee Hite, Deanna Berg

**Affiliations:** 1 Wegmans School of Pharmacy, Rochester General Hospital, Victor, New York; 2 Rochester General Hospital, Rochester, New York; 3 Rochester Regional Health, Rochester, NY; 4 Wegmans School of Pharmacy, Rochester Regional Hospital, Penfield, New York

## Abstract

**Background:**

Traditional methods in blood culture analysis require 24-72 hours to yield identification (ID) and antimicrobial susceptibility testing (AST) results, which may contribute to the use of empiric broad-spectrum antibiotic therapy. Hence, the primary objective of this study was to determine the impact of rapid blood culture analysis with the Accelerate Pheno™ system (AXDX) on time to antibiotic de-escalation.

**Methods:**

This was a single center, case-control analysis of adult inpatients with *E. coli* or *Klebsiella* spp. bacteremia. Cases were prospectively identified by the antimicrobial stewardship team between August and October 2020 after the implementation of AXDX in July 2020. Subjects were matched to historical controls (July 2018-July 2020) based on age (± 3 years), gender, source of infection, and identified organism. The primary outcome was time to antibiotic de-escalation and time to oral antibiotic therapy from the time of positive blood cultures. Secondary outcomes included hospital length of stay, 30-day mortality, 30-day readmission, and 60-day *C. difficile* infection. Outcomes were compared using descriptive and inferential statistics.

**Results:**

Of 33 cases identified, 30 (91%) were matched with historical controls. *E. coli* bloodstream infection was identified in 24 (80%) subjects while *Klebsiella* spp. was identified in 6 (20%) subjects. The average age was 66 years (SD ± 19) and there was an even distribution of males and females in both groups. Other demographics were similar between groups. The median time to species identification [14 hours (IQR 13 – 18) vs 34 hours (29 – 39), p< 0.001) and AST [20 hours (19 – 37) vs 45 hours (38 – 51), p< 0.001] from laboratory registration was significantly shorter in cases. The average time to antibiotic de-escalation was 1.7 (±1.2) days for cases compared to 2 (±1.3) days for controls (p=0.460). Median time to oral antibiotic therapy from positive blood cultures was 2.9 (1.8 – 4.7) days for cases and 3.4 (2.5 – 5.1) days for controls (p=0.166). There were no significant differences in the secondary outcomes.

**Conclusion:**

AXDX did not appear to have a significant impact on time to antibiotic de-escalation and time to oral antibiotic therapy. However, time to organism ID and AST results were significantly shorter in the AXDX cohort.

**Disclosures:**

**All Authors**: No reported disclosures

